# Sustainable and Ecofriendly Chemical Design of High Performance Bio-Based Thermosets for Advanced Applications

**DOI:** 10.3389/fchem.2021.691117

**Published:** 2021-05-28

**Authors:** Mehdi Derradji, Oussama Mehelli, Wenbin Liu, Nicholas Fantuzzi

**Affiliations:** ^1^ UER Procédés Energétiques, Ecole Militaire Polytechnique, Algiers, Algeria; ^2^ Institute of Composite Materials, Key Laboratory of Superlight Material and Surface Technology of Ministry of Education, College of Materials Science and Chemical Engineering, Harbin Engineering University, Harbin, China; ^3^ DICAM Department, University of Bologna, Bologna, Italy

**Keywords:** polymers (A), green chemistry, thermosets, biomaterials, composite

## Abstract

High performance thermosetting resins are targeted in many exigent applications, such as aerospace and marine fields, for the development of lightweight structural composites. Till now, these industries only rely on petroleum-based materials for their supposedly better performances. However, the latest developments in the field suggest otherwise. In fact, many reports confirmed that sustainable and ecofriendly thermosetting polymers can display similar or even better performances. Additionally, exploring alternative renewable feedstock’s to meet the ever increasing demands of these industries is an essential step towards sustainable development. Aiming to unravel the potential of these materials, the present review summarizes the most relevant chemical routes allowing the preparation of fully or partially bio-based thermosetting resins. Meanwhile, the overall performances of these exceptional materials are also compared with their petroleum-based counterparts.

## Introduction

Since World War II, lightweight polymeric materials with metal-equivalent properties are needed to replace heavy and expensive metallic parts in different industries. However, only few developed polymers can meet these exigent requirements. High performance thermosetting resins, which are key players in the advanced materials sector, are one of these few. Owing to their high modulus, strength, durability, and thermal and chemical resistances, these polymers provide high performance products for industry. The most commonly used polymers are epoxy, bismaleimide, benzoxazine, and phthalonitrile, especially in aerospace, microelectronic, sporting products, as well as automobiles industries. The synthesis processes of these materials, similarly to other polymers, have been exclusively relying on petroleum based reagents. However, the dwindling of fossil resources along with the increase of the polymer consumption have encouraged research interests in alternative and bio-based performance thermosets. Additionally, the massive use of petroleum-based polymeric materials has led to potential dangerous wastes, causing subsequent environmental concerns. Therefore, exploring the use of chemicals from renewable resources to synthesize bio-based thermosets is more than necessary in order to meet the requirements for sustainable development.

The path toward much greener and stronger bio-polymers has been set some twenty years ago by Anastas and Warner (Anastas and Warner, 1998). Indeed, these talented researchers provided guidance and footprints for future generations to achieve the targeted goals of “Green Chemistry.” Based on these principles, sophisticated approaches to overcome these barriers could include the use of (i) renewable feedstock (e.g. biomass residues); (ii) neoteric solvents (e.g. ionic liquids (IL) or polyethylene glycol (PEG)); and (iii) more energy-efficient heating sources, like microwave irradiation. It is important to highlight that the use of neoteric solvents can be avoided if non-reactive and fully recycled conventional solvents, such as toluene, are used during the reaction. Ultimately, a combined approach remains the only way to achieve fully-bio polymers, while other combinations will only result in partially-bio materials. However, the term of fully-bio monomers can be used only by combining the two first approaches. Obviously, the previous classifications are not intended to undermine any effort; however, a proper nomenclature should be respected for scientific rigor.

The use of renewable feedstock has been the main explored area due to the exponentially growing availability of the materials. In fact, many industrials reagents suppliers are now selling these products along with the petroleum based ones, hence providing easy worldwide accessibility to these materials. Considering that the conditions that are used in synthesizing a polymer can have a significant effect on the pathway’s overall environmental impact, a great deal of the environmental concern is associated with chemicals coming from substances associated with their manufacture. The most visible of these associated substances are the solvents used in reaction media, separations and formulations. Many solvents, especially the widely used volatile organic solvents, have come under increased scrutiny and regulatory restriction based on concerns for their toxicity and their contributions to air and water pollution. It is for these economic and environmental reasons that much of the research and development in Green Polymer Chemistry reaction conditions is focused on alternative solvents. When it comes to replacing the traditional solvents, the use of neoteric solvents may be a good alternative depending on the reaction conditions. Interestingly, some thermosets synthesis processes have reached a great maturity and in some cases the solvents are not even required. In order to complete the transformation from monomers to polymers, high curing temperatures are generally applied to move forward with the curing process. Completing the path of fully-green materials, more energy-efficient heating sources such the microwave radiation can be sometimes applied, depending on the nature of the monomers.

Overall, this report provides an up-to-date review about the design of sustainable and ecofriendly high performance thermosets. The review focuses on three thermosets, namely benzoxazine, phthalonitrile, and bismaleimide, which have been proved to be promising building blocks for composite structures with metal or ceramic equivalent performances. The synthetic routes are thoroughly discussed and the obtained monomers and polymers are compared with their petroleum based counterparts. This review also aims to unravel the potential of these green materials and to highlight the areas that need further investigations.

## Benzoxazine Resins

**FIGURE 1:**
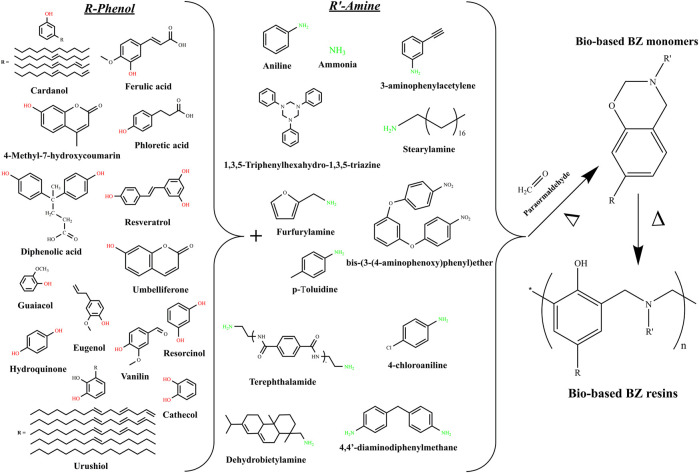
Synthetic routes of bio-based benzoxazine monomers.

Benzoxazine (BZ) resins, as one of the newest high performance thermosetting resins, have attracted much of the scientific and industrial attentions in the last few years. Indeed, the BZ resins, owing to their extremely simple synthesis process and to their outstanding combinations of extremely valuable properties, performed to fastest jump from academia to the industrial field. The interesting features of the BZ resins include a catalyst-free polymerization, low melt-viscosity, high thermal stability, good mechanical properties, low dielectric constant and low surface free energy. The BZ monomers can be readily synthetized *via* Mannich condensation reaction involving a mixture of phenols, amines and formaldehyde. The ease of synthesis and the existence of a various natural phenolic and amino derivatives may allow researchers to explore many combinations of partially and fully bio-based BZ monomers. The studied and possible combinations of bio-based BZ are displayed in Figure 1.

The first report dealing with bio-based BZs was published in 1999 by Kimura et al. (Kimura et al., 1999). The partially bio-based monomers were synthetized by reacting terpenediphenol, formaline, and aniline. The terpenediphenol-based BZ monomers were cured in the presence of epoxy and the thermal and mechanical properties were investigated. Overall, it was confirmed that the cured polymers had better heat resistance and mechanical properties compared to the bisphenol A based epoxy resin. Although this first attempt did not achieve fully green monomers and polymers, it was seen as the first spark that initiated an exponentially growing number of investigations. The following years have shown various attempts to substitute petrochemicals by bio-based products such diphenolic acid (Zúñiga et al., 2011), cardanol (Calò et al., 2007), urushiol (Xu et al., 2012), and coumarin (Kiskan and Yagci, 2007). Unfortunately, these synthetic procedures only allowed the preparation of partially bio-BZ monomers, since petroleum-based amines were still used as co-reactants. Thus, it was not until 2012 that Wang and coworkers reported about the first fully-green monomers synthesized from guaiacol, furfurylamine, and stearylamine (Wang et al., 2012), following the well-known solventless method, initiated by Ishida (Ishida, 1996), which can be applied to prepare either traditional or bio-based BZ monomers. It is important to point out that the solvent-less synthesis method is a convenient, cost competitive and environmentally friendly method for the preparation of various types of benzoxazine monomers and it is also the main reason behind the successful introduction of the BZ resins into the market. The curing behavior investigations revealed that the introduced furan moieties actively participated in the ring-opening polymerisation and accelerated the curing process. Additionally, the major improvements in the crosslinking density were afforded thanks to the reactions of the furan ring during the polymerization. Indeed, in contrast with the typical Bisphenol A-aniline based BZ (BPA-BZ) polymer, the studied bio-based polymers displayed improved glass transition temperature (T_g_) along with higher thermal stability and char yield. Thus, it was for the first time ever confirmed that bio-BZ polymers could effectively compete with their traditional petroleum based counterparts. Sini et al. also reported on the synthesis of novel fully bio-based BZ monomers prepared from of vanillin, furfurylamine, and formaldehyde using the solventless method (Sini et al., 2014). In terms of curing behavior, it was noticed a lower curing temperatures due to the presence of ACHO group in the backbone structure. On the other hand, the available report did not deal with the thermal and mechanical performances of the cured samples. In fact, one objective of this review is to highlight the areas that need further investigations.

The solventless method was also applied by Thirukumaran and coworkers to synthesize two fully-bio BZ monomers from naturally occurring sources, i.e., eugenol, furfurylamine, stearylamine, and paraformaldehyde (Thirukumaran et al., 2014). Both monomers and their copolymers showed wider processing windows and their thermal and mechanical properties were enhanced as the furan ring improved the crosslinking degree. Another interesting study was conducted by Zhang et al. in which two fully renewable BZ monomers containing multi-oxazine rings were afforded by reacting vanillin, guaiacol, octadecylamine and furfurylamine (Zhang et al., 2015). The thermogravimetric results indicated that the prepared thermosets had good thermal stability and high char yields. Meanwhile, the thermomechanical properties investigated by dynamic mechanical analysis (DMA) revealed a *T*_g_ of 155°C. In contrast with the BPA-BZ, which is characterized by a *T*_g_ of about 177°C, the studied thermosets showed lower performances. Herein, it should be reminded that the thermomechanical properties are also highly dependent on the adopted curing program. Overall, the materials reported by Zhang et al. can surely be seen as high performance thermosets.

The last year also showed a growing interest in the design of new bio-based BZ monomers and polymers. For instance, Hao et al. prepared partially bio-based BZ monomers from apigenin and furfurylamine (Hao et al., 2020). The adopted synthetic route although not fully bio afforded BZ monomers with a unique thermal latent polymerization characteristic due to the presence of intramolecular hydrogen bonds within the molecular structure. Interestingly, the cured polymers displayed an exceptionally high *T*_*g*_ of about 376°C and excellent thermal stability confirmed by a char yield at 800°C of around 66%. Zhu and coworkers followed the solventless approach to synthetize fully bio-based BZ aiming monomers having low onset curing temperature (173°C) (Zhu et al., 2020). The novel synthetic procedure involved the use of renewable amino acid derived from furfurylamine and levunilic acid. Although the authors thoroughly the curing process and kinetics, no data were provided regarded the thermomechanical properties of the cured polymers. Following the same vision, Yang et al. reported about a new tri-furan functional fully bio-BZ resin (Yang et al., 2020). The starting materials included guaiacol, furfural, furfurylamine, and paraformaldehyde. The afforded polybenzoxazine showed a *T*_*g*_ of about 290°C and a char yield at 800°C of around 62%. It is also interesting to point out that the all of the previously discussed bio-based BZ monomers used various phenolic and amine precursors, while the aldehyde part of the oxazine ring exclusively originated from the paraformaldehyde. Although these BZ monomers were referred to sometimes as fully bio-based by the authors, it should be noted that only two of the three raw materials issued from renewable resources. After an extensive literature survey, it appeared that only one study overcame this issue by substituting the paraformaldehyde with benzaldehyde. Thus, Machado et al. described the solventless synthesis of so-called truly bio-based BZ monomers from sesamol, furfurylamine, and benzaldehyde (Machado et al., 2021). The corresponding polymer has high thermal stability with 5 and 10% weight-loss temperature of 317 and 332°C, respectively, char yield of 46%, and heat release capacity of 201 J/g.k. Meanwhile, all the previously discussed BZ polymers still cannot be seen as fully green thermosets because of the chosen curing procedure.

Indeed, it has been established that the in order to achieve fully green thermosets, the curing step needs to be achieved through ecofriendly heating sources such as the microwave irradiations. Following these guidelines, Kotzebue et al. reported about fully-green BZ thermosets synthetized from two lignocellulose-based bio-products, catechol and furfurylamine, and having polyethylene glycol (PEG) as solvent (Kotzebue et al., 2018). The main drawback of these newly developed monomers was the relatively short processing window which may lead to manufacturing issues and porosities formation. Therefore, the authors provided an alternative solution based on the blending technology. In terms of thermal resistance, both polymers as well as their cured blends showed excellent thermal behavior with a degradation temperature at 5% weight loss (*T*_5%_) over 361°C and char yields at 800°C greater than 46%. The thermomechanical properties investigated by DMA highlighted initial storage moduli and glass transition temperatures (*T*_gs_) far more important than those of the BPA-BZ.

Overall the field of high performance bio-based BZ thermosets is rapidly growing and the available data are already proving better performances than the fossil-based BZs. However, a lack of proper investigations on the rheological behavior of these bio-based monomers needs to be dealt with in order to assess the compatibility of these resins with natural or traditional reinforcing phases.

## Phthalonitrile Resins

**FIGURE 2:**
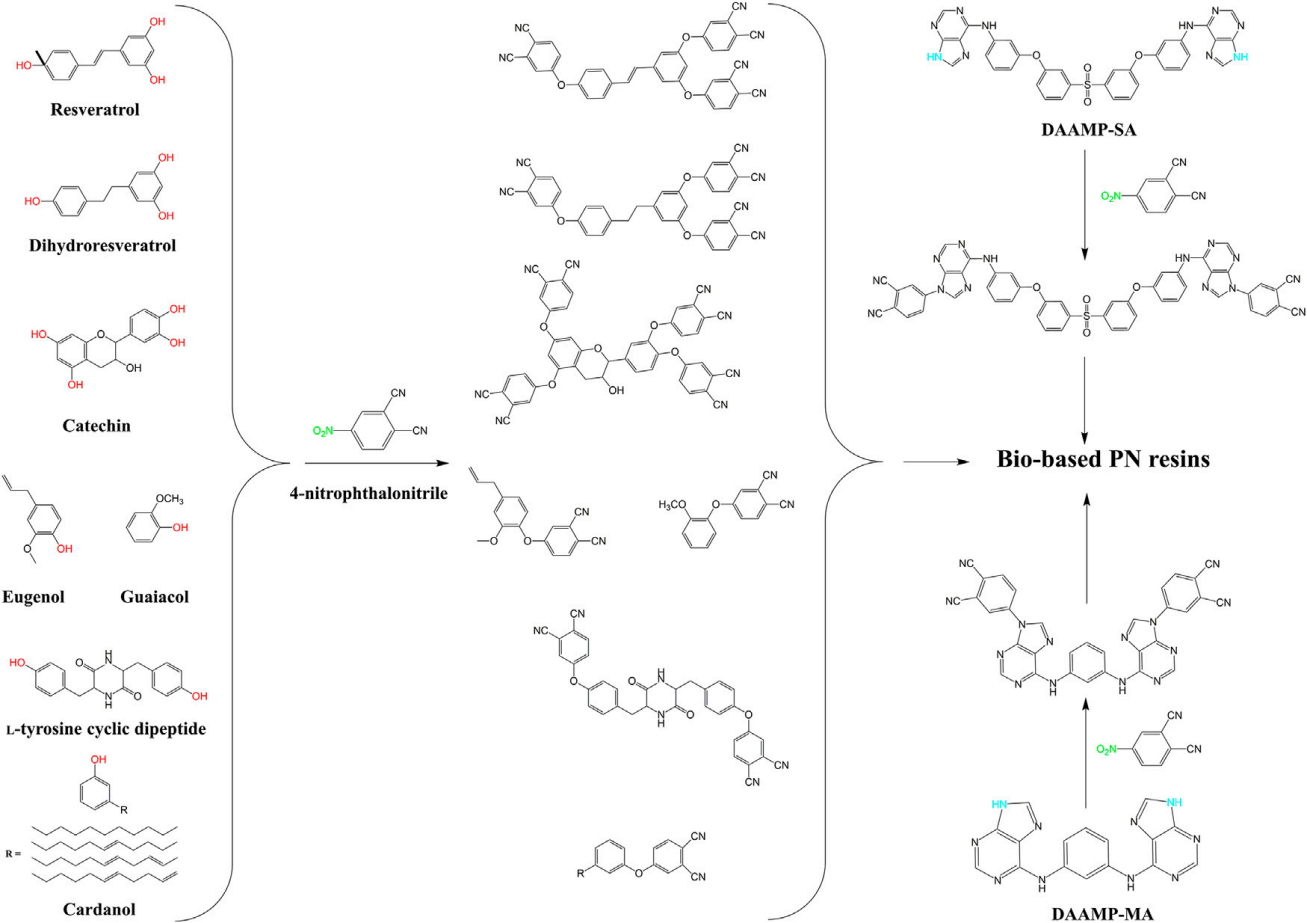
Synthetic routes of bio-based phthalonitrile monomers.

Due to their outstanding features, high-performance thermosets have been widely used in almost all sectors. Phthalonitrile (PN) resins stand on the leading edges of high performance thermosets and can be referred to as the best heat resistant polymer (Derradji et al., 2018). Similarly to the BZ monomers, the PN ones have also been mainly synthetized from fossil-based precursors. Indeed, the typical bisphenol A-based PN (BAPN), which made the success of this kind of thermosets, entirely relies on petroleum reagents. Over the last few years, there have been increased concerns about the environment which led the researchers to substitute petroleum-based PN resins, with much greener ones. The objective was to develop partially bio-based PN polymers retaining the outstanding thermal stability of their petroleum-based counterparts. These proposed alternative substitutes, summarized in Figure 2, are also based on the typical nucleophilic displacement reaction, occurring between the nitro (NO_2_) group of the 4-nitrophthalonitrile and the free phenolic hydroxyl groups (OH).

Laskoski et al. prepared the first partially bio PN monomers, based on renewable triphenols, resveratrol and dihydroreseveratrol molecules (Laskoski et al., 2016). These bio-monomers displayed wide processing windows along with an adequate rheological behavior for the preparation of advanced composites. The polymerization of these monomers resulted in the production of highly crosslinked polymers not displaying any glass transition in the interval of measure (up to 450°C). Meanwhile, the thermogravimetric measurements showed that these bio-based PN polymers were as thermally resistant as their petroleum-based counterparts, with starting decomposition temperatures over 500°C. In addition to their excellent thermo-oxidative properties, the newly synthesized PN polymers had low dielectric constant and negligible water absorption. Thus, it was concluded that these partially bio PN polymers could easily be used in the preparation of advanced structural composites for exigent applications.

In another study, Qi et al. compared the petroleum-based BAPN resin with a partially bio PN resin from a naturally occurring phenol and antioxidant moiety, namely the catechin (Qi et al., 2018). In fact, the reaction between 4-nitrophthalonitrile and catechin can potentially yield many products that are formed from a different number of phenolic hydroxyl groups reacting with nitro groups at different positions. The authors reported that both resulting polymers showed approximatively the same thermal resistance behavior with a slight advantage for the bio-based one. In other words, the latter polymer and the P(BAPN) retained 95% of their initial weight at 520 and 484°C, respectively. In fact, the catechin-based PN polymer showed higher thermal stability with lower melting point and curing temperatures than those of the conventional PN resin. Besides, after a kinetic study, the curing reactions of both resins systems undergo an autocatalytic mechanism.

Using eugenol and guaiacol extracted from clove and lignin, respectively, as precursors, Wang et al. prepared two new bio PN resins (Wang et al., 2018). This study indicates that the bio PN polymers hold better thermal and mechanical properties as well as higher glass transition temperature than that of the typical BAPN polymer. These interesting findings can be explained by a structure with no voids and a homogenous continuous phase confirmed by the morphological analyses. In another report and aiming to enhance the bio PN monomers, Peng et al. studied the feasibility of introducing a protein moiety, L-tyrosine, to prepare L-tyrosine cyclic dipeptide as a building block for the synthesis of a new bio-based PN monomer (Peng et al., 2018). After thermal, thermomechanical and kinetic studies, the new bio-resin showed excellent thermal property with high glass transition temperature compared with the traditional petroleum-based PN resins. Once again, the curing process of the bio PN resin followed an autocatalytic mechanism. These results confirmed the feasibility of introducing the L-tyrosine moiety as a bio-based precursor for the preparation of a new green thermoset with good thermal and thermomechanical properties. Later on, Chen et al. studied the impact of long alkyl chains of cardanol for the preparation of a bio-based PN monomer (Chen et al., 2019). This study showed that the incorporation of this molecule leads to a PN resin with lower room temperature storage modulus, but with greater thermal properties and glass transition temperature similar to those of conventional PN polymers. Moreover, the obtained bio-polymers did not undergo any great change after 100 kGy dose of Co^60^ irradiation. In an earlier study and contrary to mentioned works, which rely on biomolecules containing free phenolic hydroxyl groups, Liu et al. proposed a new high-performance resins based on two different diamine-adenine-monomer [flexible diamine 3,3′-(sulfonylbis 4,1-phenyleneoxy)]bis-benzenamin (DAAMP-SA) containing sulfone and aromatic ether linkages, and rigid m-phenylenediamine (DAAMP-MA) (Liu et al., 2019). The synthesized monomers presented self-curing polymerization behavior. The new diamine-based PN polymers showed different thermal polymerization behavior, with superior glass transition temperature, high thermal and thermo-oxidative stabilities. Likewise, in comparison with petroleum-based PN resin and other thermosets, DAAMP-SA-based PN polymer showed prominent thermal stability and excellent thermomechanical properties.

During the last year, some interesting studies were also published confirming the great potential of these thermosetting materials. For instance, vanillin was introduced, by Han et al. (Han et al., 2020) and Wang and workers (Wang et al., 2021), as a sustainable source for PN monomers design. Both group of researchers reported about the synthesis and properties investigations of vanillin-based PN monomers and polymers. The obtained result revealed that the cured polymers had *T*_*gs*_ over 400°C and char yields at 800°C around 76%. Magnolol derivatives were also used by Weng et al. to develop new bio-based PN monomers and polymers (Weng et al., 2021). The afforded monomers displayed outstanding processability with a processing window of 163°C and excellent thermal resistance with a *T*_*g*_ over 500°C and starting decomposition temperatures over 527°C.

Overall, the field of bio-based PN is limited to partially bio-monomers and polymers and much work needs to be performed to find a proper way to synthetize fully bio PN resins. Therefore, this area of research can be seen as an opportunity to widen the panel of high performance bio-thermosets and extend their applications.

## Bismaleimide Resins

**FIGURE 3:**
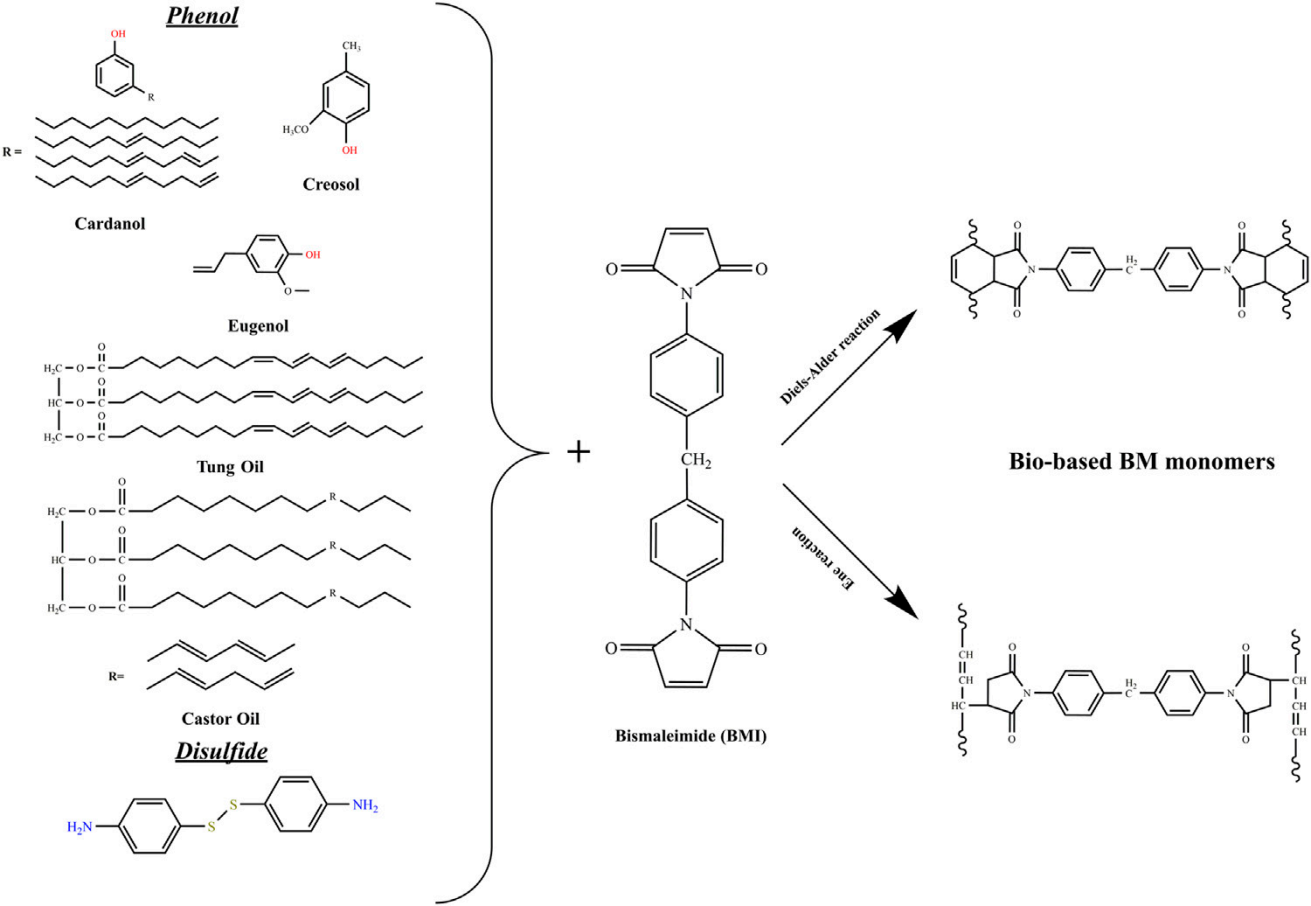
Synthetic routes of bio-based bismaleimide monomers.

As stated previously, thermosets derived from renewable feedstock are gaining more interest in the last years. Indeed, scientists are trying to substitute petroleum products with green compounds in all fields, including polymers production. Among these polymers, polybismaleimides, which are thermosetting with high performances (high strength and rigidity, excellent electrical properties, etc.). Generally, polybismaleimides are obtained by copolymerization of bismaleimide monomers with 2,2′-diallyl bisphenol A (DBA), which follows a two reactions mechanism, namely Diels-Alder (DA) and/or ene reactions (Iredale et al., 2017). Many efforts, as seen in Figure 3, were dedicated to substitute the petroleum component (DBA) in the polymerization process. For instance, Hirayama et al. copolymerized Dehydrated Castor Oil (DCO) and bismaleimide (BMI) (Hirayama et al., 2009), and in a similar study, Shibata et al. used Tung Oil (TO) instead of DCO (Shibata et al., 2011a). The cured resins also followed both the DA and ene reaction mechanisms. The analysis revealed that tensile modulus, glass transition and 5% weight loss temperatures were improved upon the BMI content increase. Meanwhile, the tensile strength highest value was obtained with a DCO/BMI ratio of 1/1. Similarly, the glass transition and 5% weight loss temperatures, augmented when BMI content increased in the TO/BMI resins, and the tensile strength and modulus highest values were recorded with a TO/BMI ratio of 1/2 and 1/3, respectively. In a later study, Shibata et al. elaborated bio-based polybismaleimide using cardanol novolac (Shibata et al., 2013a). They first synthesized the monomers using cardanol, paraformaldehyde and oxalic acid, as solvent. Then, different monomers were prepared using cardanol and cardanol novolac with 4,4′-bismaleimidediphenylmethane at different ratios, followed by the curing reaction to provide fully cured polymers. Nuclear Magnetic Resonance (NMR) analysis of the resins implied that the ene reaction was predominant followed by DA reaction. While the FTIR analysis suggested that both ene reaction and addition copolymerization were the predominant reaction mechanisms. The thermal and mechanical analysis revealed that the *T*_g_ of the CD/maleimide unit with ration lower than ½ did not appear till 300°C. Interestingly, the 5% weight loss temperature was higher than 450°C and the best flexural strength and modulus were measured at a CD/maleimide unit ratio of ¼.

Eugenol (EG) is an aromatic compound found mainly in cloves. It can also be extracted from peppers from Jamaica, bay leaf and cinnamon known as cloves. This natural product has been widely used as an alternative to petroleum products in the preparation of bio-thermosets, including the polybismaleimides. Shibata et al. used succinic acid and EG as main products to provide high-performance green bismaleimide resins (Shibata et al., 2011b). They first prepared biseugenyl succinate (BEUS) and bis(4-maleimidephenyl) succinate (BMIS). Then, they elaborated and compared the cured resins (mixtures of BEUS/BMIS at different molar ratios) and 2,2′-diallyl bisphenol A (DABA)/4,4′-bismaleimidodiphenylmethane (BMIM). The FTIR spectral analysis revealed that chain polymerization of allyl and maleimide groups, was the main reaction mechanism in BEUS/BMIS polymerization, while ene, chain polymerization and etherification reaction mechanisms occurred to produce DABA/BMIM resins. Besides, the thermomechanical analysis showed that BEUS/BMIS resins with ratios of 1/2 and 1/3 had the highest glass transition temperature and tensile strength than that of DABA/BMIM resins. Further, Shibata and coworkers synthesized 5,5′-Bieugenol (BEG) and eugenol novolac (EGN) from EG and then produced cured bismaleimide resins by reacting EG (reaction 1), BEG (reaction 2), and EGN (reaction 3) with BMIM at EG/maleimide different unit ratios (Shibata et al., 2013b). FTIR and NMR spectral analysis showed that reaction 1 main model reactions were the ene reaction and subsequent Diels-Alder/ene reactions. While the second and third reactions main models were the ene reaction and subsequent thermal addition copolymerization. The thermomechanical analysis reported that glass transition and 5% weight loss temperatures increased with BMIM content augmentation, and resin of reaction 1 showed the highest values. Regarding mechanical properties, flexural strength and modulus of resins from reaction 1 and 3 were higher than those from reaction 2, with the best values of cured EG/BMIM 1/2. Neda et al. presented a similar study in which they prepared allyl-etherified EG and BEG (AEG and ABEG), then the cured resins by reacting AEG and ABEG with BMIM resins with allyl/maleimide different unit ratios (Neda et al., 2014). The thermal and mechanical properties were higher than those reported in the Shibata et al. previously described works.

Cardanol, one of the inexpensive natural resources, also has been used to prepare bio-based BMI compounds. Hannoda et al. used it to, first, synthesize cardanyl linolenate (CDLN) and allyl cardanyl ether (ACDE) and then reacting them with BMIM to produce cured resins at different molar ratios (Hannoda et al., 2015). The FTIR spectral analyses showed that the addition copolymerization or ene reaction mechanisms occurred during the curing process. In terms of performances, the thermomechanical tests revealed that cured ACDE/BMIM resins had higher 5% weight lost temperature and flexural properties than that of cured CDLN/BMIM resins. In a recent report, Ge et al. also reported about a newly developed bio-based allyl ether compound, bis (5-allyloxy)-4- methoxy-2-methylphenyl methane (ABE), synthesized from renewable lignin derivate creosol. The thermosetting bio-resins were found to display better processability, high T_g_ and mechanical properties than the conventional petroleum-based BMI resins (Ge et al., 2018).

Interestingly, in a newly published research by Lejeail et al. suggested the replacement of the typical BMI by a bio-based bisitaconimide *via* thermoreversible Diels-Alder (DA) cross-links (Lejeail and Fischer, 2020). It should be herein highlighted that itaconic acid-based thermosetting resin are exponentially gaining interest in the field of bio-polymers. Indeed, itaconic acid, which is an economically viable bio-renewable building block produced by the fermentation of biomass such as corn, rice, or lignocellulosic feedstocks, can used as a starting materials to develop greener thermosetting resins (Trotta et al., 2018). Meanwhile, the recent developments in the field of itaconic acid-based polymers suggest the possibility to develop poly (itaconic acid) polymers with exceptional properties (Jahandideh et al., 2018a; Jahandideh et al., 2018b).

BMI based bio-polymers can also regarded as a promising materials for the replacements of their fossil based counterparts. Based on the available data, we are expecting a rapidly growing number of researches on the field of bio-composites based of BMI matrices. It is also crucial to point out that similarly to the PN resins, the path toward fully green BMI monomers is still an area that requires many efforts. Indeed, it is one goal of this small review to highlight the area that needs further investigations.

## Conclusion

This effort provided a multifaceted review of the latest advances on the synthesis of high performance bio-thermosets intended to be used in extreme environmental conditions. This review thoroughly detailed the chemical design and the resulting properties of three leading and well-known high performance thermosets, namely the benzoxazine, phthalonitrile, and bismaleimide polymers. The synthetic routes and procedures were classified according to the rules of the “Green Chemistry”. The available data suggest that only benzoxazine polymers have fulfilled the requirements of fully green materials, whereas further efforts are needed for the phthalonitrile and bismaleimide thermosets to reach this stage. In terms of performances, it has been confirmed that, in almost all cases, the bio-thermosets displayed similar or better performances than their petroleum-based counterparts. Overall, this contribution aimed at revealing the huge potential of these biomaterials in the global vision of sustainable and ecofriendly design of high performances materials.
